# Omega-3 Fatty Acids Supplementation in Children to Prevent Asthma: Is It Worthy?—A Systematic Review and Meta-Analysis

**DOI:** 10.1155/2015/312052

**Published:** 2015-08-19

**Authors:** Prasad Muley, Monali Shah, Arti Muley

**Affiliations:** ^1^Department of Paediatrics, S.B.K.S MIRC, Sumandeep Vidyapeeth, Vadodara, Gujarat 391760, India; ^2^Department of Periodontics, K.M.S DCH, Sumandeep Vidyapeeth, Vadodara, Gujarat 391760, India; ^3^Department of Medicine, S.B.K.S MIRC, Sumandeep Vidyapeeth, Vadodara, Gujarat 391760, India

## Abstract

Asthma is one of the most common respiratory diseases affecting all age groups. The world is now trying to identify some dietary factors which can play a preventive role. We performed this systematic review and meta-analysis of RCTs to assess the effect of intake of polyunsaturated fatty acid (PUFA) in infancy and/or childhood on incidence of asthma or wheezing episodes. We searched MEDLINE, EBSCO, Trip, and Google Scholar up to January 31, 2015. All RCTs where infants or children who were given omega-3 fatty acid supplementation and which reported incidence of asthma and/or wheezing episodes as dichotomous outcomes were included in this review. Random effects model was used for pooling the risk estimates. Total five articles were included. Most of them were from Australia. On meta-analysis, the pooled estimate of odds ratios by random effects model showed no significant change in incidence of asthma after supplementation of omega-3 FA in infancy or childhood (OR 0.974; CI 0.646, 1.469; *p* = 0.900). We concluded that a multicentric RCT is required to assess the effect of omega-3 FA supplementation exclusively to infants or children to predict the best time of omega-3 FA supplementation to prevent asthmatic or wheezing episodes later in life.

## 1. Introduction

Asthma is one of the most common respiratory diseases affecting all age groups. Around 300 million people are suffering from this disease worldwide [1]. The increasing prevalence of asthma over the years is an important cause of concern all over the globe. Inhaled corticosteroids are used for the management of asthma, but 5–10% of asthma patients are resistant to this therapy, leading to difficulties in managing the disease [2]. Hence, the world is now focusing on environmental factors and genetic susceptibility to identify the reason for this surge so that some effective strategy to prevent the disease can be developed. For the same reason, there has been increase in studies to find some dietary factors which can play a preventive role.

Omega-3 fatty acids are essential nutrients that cannot be synthesized in the body and must be obtained from the diet. Dietary omega-3 fatty acids are incorporated into cellular membranes of all tissues and are naturally enriched in fatty fish like salmon and tuna and in fish oil supplements. There have been reports that omega-3 fatty acids (omega-3 FA), docosahexaenoic acid (DHA), and eicosapentaenoic acid (EPA) have protective role against coronary artery diseases, hypertension, and dyslipidemia [3]. Their preventive role has also been reported in diabetes [4] and chronic inflammatory diseases including chronic obstructive pulmonary disease (COPD), rheumatoid arthritis, and inflammatory bowel disease [5, 6]. Molecular mechanisms have been revealed to support efficacy of omega-3 fatty acids in prevention of asthma and allergic diseases in part by the identification of fatty acid bioactive metabolites. It has been proposed that in severe asthma specialized proresolving mediators (SPM: protectins, resolvins, and maresins) production is impaired. Thus, by generating SPM, omega-3 fatty acids such as EPA and DHA counterregulate airway eosinophilic inflammation and promote the resolution of inflammation in vivo [7].

Fish oil contains two main omega-3 fatty acids, docosahexaenoic acid (DHA) and eicosapentaenoic acid (EPA). Many epidemiological studies have reported reduction in incidence of allergic or atopic outcomes in infants or children of those pregnancies as an effect of maternal fish intake during pregnancy [8–12]. Some questionnaire based studies have also reported a protective effect of fish intake during infancy or childhood on allergic outcomes [13–19] paving the way for the question whether long-chain omega-3 FA have any significant role in prevention of asthma. A systematic review and meta-analysis assessed the relation of omega-3 FA supplementation (both in mothers and children) with the incidence of asthma or allergic or wheezing episodes; however it did not include RCTs [20]. So, we performed this systematic review and meta-analysis of RCTs to assess the effect of intake of polyunsaturated fatty acid (PUFA) in infancy and/or childhood on the incidence of asthma or wheezing episodes. Our research question was “In children (P) does omega-3 fatty acid supplementation (I) as compared to no intervention (C) help in primary prevention of asthma (O)?”

## 2. Methodology

### 2.1. Searching

A comprehensive search was performed using search terms (Omega 3 fatty acids OR PUFA OR fish oil) AND (Asthma OR wheezing episodes). We used medical subject headings (MeSH or MH) as search terms when available or key words when appropriate and searched four electronic databases, namely, MEDLINE, EBSCO, Trip, and Google Scholar up to January 31, 2015. Limits used were “humans,” “children,” and “0–18 years.” We searched only for RCTs. Additional information was retrieved through hand search of the references from relevant articles. Three authors independently reviewed all titles retrieved and abstracts were screened where title was unclear. The abstracts thus found relevant were selected for full text reading. The articles selected for full text reading were read by the three reviewers independently for assessing the risk of bias and retrieving the information relevant for the review and meta-analysis. All discrepancies were solved by group discussion.

### 2.2. Study Eligibility Criteria

Inclusion criteria were all RCTs where only infants or children were given omega-3 fatty acid supplementation and which reported change in FEV1 or asthma severity scores or incidence of asthma and/or wheezing episodes as dichotomous outcomes. Exclusion criteria were all non-RCTs, studies where mothers were also given experimental supplementation, and studies which did not report the outcome in terms of incidence of asthma or wheezing episodes after supplementation.

### 2.3. Data Extraction and Assessment of Quality of Trial

Data was extracted individually by two authors (Prasad Muley and Arti Muley) and any disagreement was resolved by opinion of the third author (Monali Shah). The details of place, sample size, age, intervention, comparator, follow-up, and outcome monitored were recorded from included studies. Risk of bias was assessed according to Cochrane assessment of risk of bias tool based on randomization, blinding, allocation concealment, loss to follow-up, and intention to treat analysis. Quality of included trials was categorized as high risk, moderate risk, and low risk if more than two, two, or one criterion were not fulfilled, respectively.

### 2.4. Statistical Analysis

We used comprehensive meta-analysis version 2 for pooling the results. We used odds ratio as the outcome measure. In the studies in which outcome was presented as relative risk or ARR (absolute risk reduction), we derived information of number of episodes of wheezing or asthma and calculated odds ratio from it to get uniform measure of outcome as odds ratio for all included studies. *I* ^2^ was used as a measure of heterogeneity. *I* ^2^ > 50% was considered significant heterogeneity. Random effects model was used for pooling the risk estimates when heterogeneity among the studies was more than fifty percent and fixed effects model when it was less than fifty percent. A sensitivity analysis was also carried out to find if there was any study which had grossly impacted the overall pooled result. We did not exclude any study on the basis of risk of bias.

## 3. Results

### 3.1. Searching

**Figure 1:**
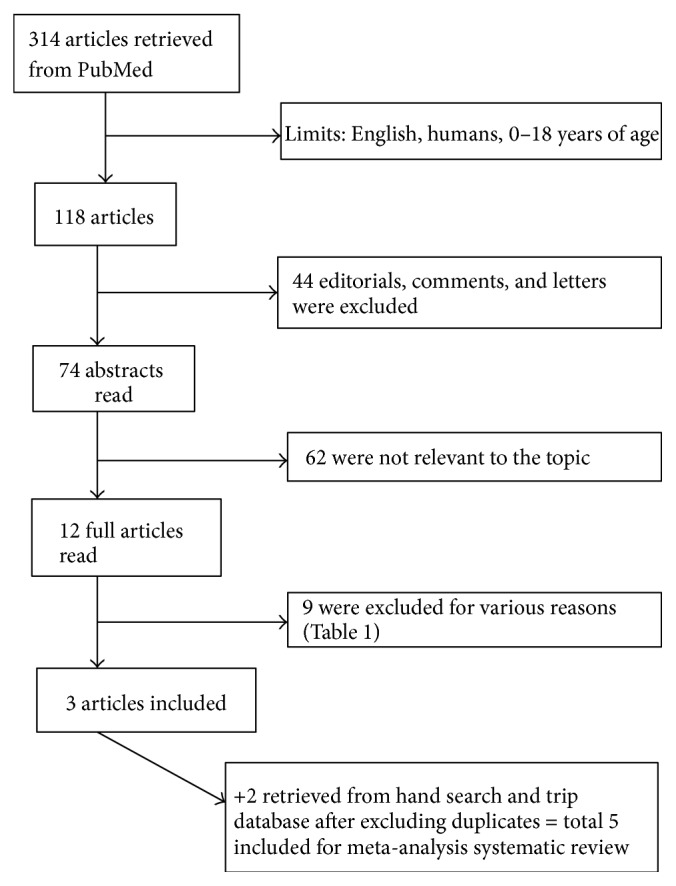
Searching details.

Fourteen relevant RCTs were identified for full text reading after comprehensive search of published literature and, thereafter, exclusion of irrelevant titles and abstracts (Figure 1). Twelve articles were retrieved from electronic database and two from hand searching. After full text reading, nine [17, 21–28] were excluded for various reasons (Table 1) while five articles were included (Table 2) in the meta-analysis [29–33].

### 3.2. Design and Quality of Included Studies (Table 3)

All the included trials reported adequate randomization and blinding while allocation concealment was done in all except one. One trial mentioned doing intention to treat analysis also. Using Cochrane tool for assessing risk of bias, one study showed moderate risk of bias while others had low risk of bias.

### 3.3. Study Characteristics

Total 2415 children were randomized in the studies and outcome was available for 1932 (80%) children. Average age of administration of supplements was 0–12 months. Longest duration of supplementation was up to five years of age in the study by Ng et al. [33]. Average duration of follow-up was 3.5 years ranging from 6 months [32] to as long as 8 years [33]. Almost all of them gave fish oil capsules as supplementation of omega-3 FA. The content of omega-3 FA was 60% in only one study [32] while the content in all other studies was about 37%. All the RCTs which reported the results as relative risk or hazard ratio or odds ratio were included in the meta-analysis.

### 3.4. Heterogeneity and Pooled Result

**Figure 2:**
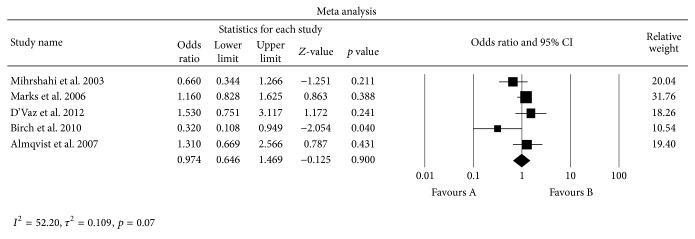
Odds ratio and 95% confidence interval of risk of asthma. The pooled estimates were obtained using a random effects model. The squares represent relative risk in each study, with square size representing the study-specific weight and the 95% CI is represented by horizontal bars. The diamond at the bottom indicates summary risk estimate.

The included studies did not show significant heterogeneity amongst them (*I* ^2^ = 52.20, Tau^2^ = 0.109, *p* = 0.07). On meta-analysis, the pooled estimate of odds ratios by random effects model showed no significant change in incidence of asthma after supplementation of omega-3 FA in infancy or childhood (OR 0.974; CI 0.646, 1.469; *p* = 0.900) (Figure 2).

### 3.5. Sensitivity Analysis

We also did sensitivity analysis to find out whether the pooled result was substantially influenced by one study. On removing one study [31] the heterogeneity reduced substantially to *I* ^2^ = 14.18, *p* = 0.316, and Tau^2^ = 0.018. On calculating pooled OR after omitting this study, the pooled result shifted slightly to right (OR 1.122; CI 0.869, 1.448; *p* = 0.378). No other individual studies significantly influenced the results.

## 4. Discussion

Some clinical trials have shown an increase in levels of omega-3 FA in the offspring with maternal intake of fish oil. They have also reported anti-inflammatory effects of using fish oil supplementation (during pregnancy and lactation) on cytokine production, lipid mediator release, and cellular infiltration [34–38]. A large epidemiological study showed an inverse association of intake of omega-3 FA with asthma morbidity. Since then, many studies focusing on omega-3 FA intake or supplements have been conducted. Most of them reported a protective effect of omega-3 FA supplementation given to mothers during pregnancy or lactation on the incidence of asthma or wheezing episodes or other allergic outcomes during infancy or childhood [8–12].

There have been some studies which assessed effect of omega-3 supplementation to infants or children. The cohort studies reported a preventive role of fish intake during infancy or childhood on allergic outcomes [11, 13–17]. The clinical intervention studies also showed a decline in prevalence of wheeze and use of bronchodilator at 18 months of age, with fish oil supplementation in infants/children of age group 6 months to 5 years [23, 29]. They also reported reduced allergic sensitization and prevalence of cough at 3 years of age. One out of two studies which examined the effect of fish oil supplements on asthmatic symptoms and lung function in children reported significant reduction in asthma severity [21, 39].

However, in adults, there have been inconsistent reports regarding benefits of fish or fish oil intake on asthmatic episodes [40–45]. Some studies have reported improved lung function and prevention of asthma with high intake of DHA [42, 43]. However, another study showed increased respiratory symptoms with reduced omega-3 FA intake [46]. This pattern was explained previously by the fact that chronic inflammation may affect the immune system [4] which has been suggested to be “particularly true in children because a child's immune system is under development” [18]. From this, it can be presumed that omega-3 FA are important in the stage of immune system development. In other words, children may be more sensitive to omega-3 FA intake than adults. A recent meta-analysis of observational studies found that “the potential beneficial effect of fish or Omega-3 FA intake was more pronounced in children. It might be explained by the suggestion that children are more sensitive to omega-3 FA.” [20].

Some other meta-analyses of RCTs have been done previously, which reported no improvements in asthmatic symptoms with omega-3 fatty acid supplementation [47, 48] both in children and adults. All included RCTs in that meta-analysis [47] had relatively small sample sizes (from 12 to 45) and short follow-up periods (from 8 weeks to 12 months). Also, fish oil was used as supplement which may reflect a different health impact from consuming whole fish, a package of nutrients [48]. Our study is different as we included only RCTs in which omega-3 fatty acids supplementation was given exclusively to children and not to mothers during pregnancy or lactation. All the studies had good sample sizes ranging from 200 to 600; the supplementation was given in the form of fish oil capsules with predetermined contents and quantities and the mean duration of follow-up was 3.5 years. A limitation of our study was that out of the five studies included in meta-analysis, three were parts of the same CAPS (Childhood Asthma Prevention Study); however, we considered them separate studies as the duration of follow-up in those was different and the data were analyzed separately in the three studies. Another peculiar observation was that four out of the five RCTs were carried out in Australia while one was from US. Hence, RCTs from other parts of the world were lacking. We also did not find any significant reduction in incidence of asthma on meta-analysis of these studies. The pooled result did not change much on sensitivity analysis but showed a drift towards right on eliminating one [31] study.

We found two studies assessing change in FEV1 [21, 26] and two studies assessing change in asthma severity scores [21, 22] after supplementation of omega-3 FA in asthmatic children. One of the studies which assessed asthma severity score used symptoms for scoring while the other used auscultatory findings for the same; hence it was not possible to pool the results. Out of those which had FEV1 as outcome measure, one study used omega-3 FA with vitamin C and Zn for supplementation [26], so pooling the results was not logical in this case either. Thus, we excluded all these from meta-analysis. However, it was noted that in these studies also there was inconsistent result regarding improvement in lung function after the supplementation. One [21] of these studies showed no significant improvement while the other two reported improved lung function with the supplementation. Although one study [26] showed improvement, it was only with combined supplementation of omega-3 FA, vitamin C, and Zn; there was no significant improvement on mono supplementation with either of them.

Thus, although many studies have reported significant reduction in incidence of asthma or wheezing episode with omega-3 FA supplementation given to mother during pregnancy or lactation, there are insignificant results with supplementation in adults. In our study, we observed lack of RCTs assessing effect of omega-3 supplementation in infancy or childhood in preventing asthmatic or wheezing episodes. The result in our study also did not show any significant reduction in incidence of asthma or wheezing episode after omega-3 supplementation exclusively in infancy or childhood. There are many theories to explain the anti-inflammatory effect of omega-3 FA but none of them appears to explain this difference, although it can be said that some substance present in maternal tissue or breast milk or some biochemical process during secretion of breast milk might be responsible. So, this study highlights the need of a multicentric RCT to find out the preventive effect of omega-3 supplementation in infancy and childhood on later asthmatic and wheezing episodes. This may answer the query of the best time of supplementation with omega-3 FA to prevent asthmatic or wheezing episodes in children.

## 5. Conclusion

We did not find any role of omega-3 fatty acid supplementation in children in primary prevention of asthma. However, there are very few RCTs available. As some studies have shown association of maternal supplementation with prevention of asthma in children, a multicentric RCT is required to assess the effect of omega-3 FA supplementation exclusively to infants or children to predict the best time of omega-3 FA supplementation to prevent asthmatic or wheezing episodes later in life.

## Figures and Tables

**Table 1:** Details of excluded studies.

Serial number	Author	Title of paper	Reason for exclusion
1	Hodge et al., 1996 [17]	Consumption of oily fish and childhood asthma risk	Case control study

2	Hodge et al., 1998 [21]	Effect of dietary intake of omega-3 and omega-6 fatty acids on severity of asthma in children	Asthma severity scoring was based on symptoms while, in other study which reported asthma scoring system, it was based on auscultatory findings, hence excluded from meta-analysis

3	Nagakura et al., 2000 [22]	Dietary supplementation with fish oil rich in omega-3 polyunsaturated fatty acids in children with bronchial asthma (asthma severity score)	Asthma severity scoring was based on auscultatory findings while, in other study which reported asthma scoring system, it was based on symptoms, hence excluded from meta-analysis

4	Mihrshahi et al., 2004 [23]	Effect of omega-3 fatty acid concentrations in plasma on symptoms of asthma at 18 months of age	Assessed association of plasma level of omega-3 FA after giving tuna fish and margarine oil with incidence of asthma

5	Chan-Yeung et al., 2005 [24]	Canadian childhood asthma primary prevention study: outcomes at 7 years of age	Did not use omega-3 FA as intervention

6	Almqvist et al., 2007 [25]	Omega-3 and omega-6 fatty acid exposure from early life does not affect atopy and asthma at age 5 years	Observational study

7	Al Biltagi et al., 2009 [26]	Omega-3 fatty acids, vitamin C, and Zn supplementation in asthmatic children: a randomized self-controlled study	Simultaneously used omega-3 FA, vit. C, and Zn supplementation

8	Manley et al., 2011 [27]	High-dose docosahexaenoic acid supplementation of preterm infants: respiratory and allergy outcomes	Supplementation given to mothers and EBM (expressed breast milk) fed to the babies

9	Lang et al., 2013 [28]	Nutrigenetic response to omega-3 fatty acids in obese asthmatics (NOOA): rationale and methods	Results not stated

**Table 2:** Details of included studies.

Serial number	Author	Year	Place	Sample size	Age/sex	Intervention and dose	Comparator and dose	Follow-up (years)	Outcomes studied	Conflict of interests	Funding
1	Mihrshahi et al. [29]	2003	Australia	616/554	6–18 months/ both	o-3–rich tuna fish oil (500 mg) from the age of 6 months.	Sunola oil	1	Number of episodes of wheeze or cough lasting for >1 week not associated with colds	None	Institute of Health

2	Marks et al. [30]	2006	Australia	616/516	6 months to 5 years	Oil capsules with 500 mg tuna fish oil (37% o-3 PUFA + 6% o-6)	Oil capsules with 0.3% o-3 + 7% o-6 PUFA	5	Episodes of wheeze, asthma	None	Institute of Health

3	Birch et al. [31]	2010	USA	147/89	<5 days–12 months/ both	DHA and ARA as 0.32%–0.36% and 0.64%–0.72% of total FA	Enfamil with iron	3	Diagnoses of wheezing, asthma	None	National Institute of Health USA

4	D'Vaz et al. [32]	2012	Australia	420/323	0–6 months/ both	650 mg fish oil capsules (280 mg DHA + 110 mg EPA) daily	650 mg olive oil (66.6% n-9 oleic acid)	0.5	Physician diagnosed IgE-mediated asthma episodes	None	National Health and Medical Research Council (NHMRC)

5	Ng et al. [33]	2010	Australia	616/450	Birth to 5 years/ both	Oil capsules, 500 mg tuna fish oil (37% o-3 PUFA + 6% o-6 PUFA)	500 mg Sunola oil capsules (0.3% o-3 + 7% o-6 PUFA)	8	ARR of wheezing episodes, asthma	None	Institute of Health

DHA: docosahexaenoic acid; EPA: eicosapentaenoic acid; ARA: arachidonic acid; PUFA: polyunsaturated fatty acid; ARR: absolute risk reduction; o-3: omega-3; o-6: omega-6; FA: fatty acids; sample size: number of participants who were given supplements/number of participants who completed the trial.

**Table 3:** Risk of bias amongst the included studies.

Serial number	Author	Randomization	Allocation concealment	Blinding	Incomplete data outcome reporting	Outcome
1	Mihrshahi et al. [29]	Randomized	Yes	Double blind	Not stated	Low
2	Marks et al. [30]	Randomized	Yes	Double blind	Not stated	Low
3	Birch et al. [31]	Randomized	Not clear	Double blind	Not done	Moderate
4	D'Vaz et al. [32]	Randomized	Yes	Double blind	Yes	No
5	Ng et al. [33]	Randomized	Yes	Double blind	Not stated	Low
